# Duckweed (*Wolffia globosa*, *Wolffia arrhiza*, and *Lemna minor*) as novel therapeutic food for acute malnutrition and famine alleviation in low- and middle-income countries and humanitarian crisis settings

**DOI:** 10.3389/fnut.2026.1835959

**Published:** 2026-09-14

**Authors:** Adriana E. Hernani-Rodrigo, Shilah F. Waters, Laura S. Valderrama Figueroa, January G. Msemakweli, Nicole LaRuta-Rosas, Marcelo Buhezo-Chamon, Robert H. Gilman

**Affiliations:** 1 Johns Hopkins Bloomberg School of Public Health, Baltimore, MD, United States; 2 Independent Researcher, Baltimore, MD, United States; 3 Departamento de Ciencias de la Salud, Universidad Católica Boliviana “San Pablo, ” Tarija, Bolivia; 4 Asociación Benéfica PRISMA, Lima, Peru

**Keywords:** acute malnutrition, duckweed, famine alleviation, food security, *Lemna minor*, therapeutic food, *Wolffia arrhiza*, *Wolffia globosa*

## Abstract

Acute malnutrition remains a leading cause of under-five mortality in low- and middle-income countries (LMICs), particularly in humanitarian crisis settings where conventional therapeutic food supply chains are frequently disrupted. There is an urgent need for locally cultivable, affordable, and nutritionally adequate alternatives to imported ready-to-use therapeutic foods (RUTFs). In this review, we examine the nutritional composition, food safety, and production scalability of three duckweed species, namely: *Wolffia globosa*, *Wolffia arrhiza*, and *Lemna minor*, as candidate therapeutic foods in famine-affected regions. Compositional analyses indicated that these species consist of a high protein content (up to 48.2, 50.9, and 39.1% dry weight for *W. globosa*, *W. arrhiza*, and *L. minor*, respectively), a comprehensive essential amino acid profile, and an array of micronutrients and immunomodulatory compounds. As indicated by a Protein Digestibility-Corrected Amino Acid Score (PDCAAS) of 89% for *W. globosa* and the Digestible Indispensable Amino Acid Score (DIAAS) of 0.75 for *W. arrhiza*, the protein quality of duckweed is considered high. Furthermore, as one of the fastest-growing plants on earth, duckweed can double their biomass in only 4–5 days, reach harvest size within 2–5 days, and produce up to ∼177 g of biomass per square meter per day, with water requirements up to 90% lower per kilogram of protein produced as compared to conventional crops. Although antinutritional factor levels are comparable to common vegetables and pathogen risks are manageable via solar drying and boiling, two primary limitations restrict their immediate application. First, a low-fat content (0.5%–9.6% DW) that confines energy density relative to standard RUTFs and, second, a susceptibility to accumulating heavy metals from growth medium. Additionally, the current evidence base may be constrained by absence of data regarding cultivation failures or adverse implementation outcomes. Consequently, rigorous, context-specific risk-benefic assessments are required. Controlled cultivation trials in LMICs, integrated with randomized controlled trials in malnourished cohorts, are necessary to objectively determine the feasibility of these species for acute malnutrition management.

## 1 Introduction

Malnutrition remains a leading cause of under-5 mortality in low- and middle-income countries (LMICs). In 2022, an estimated 148 million children were stunted, and 45 million were wasted (1). Furthermore, undernutrition remains a primary driver of global child mortality, contributing to 45% of all deaths among children under five (2). Catastrophic hunger is concentrated in humanitarian crisis settings, such as those that persist in Gaza, Sudan, South Sudan, Haiti, and Mali (3). Conditions here not only pose an immediate threat to child survival but also heighten vulnerability to infectious diseases and carry long-term consequences, as early-life malnutrition and stunting have been strongly associated with impaired cognitive development in later childhood (4).

Ongoing conflict, political instability, economic collapse, and climate-related disasters are driving mass displacement, crop failure, infrastructure destruction, and extreme weather events, leaving millions of people in severe hardship and making hunger a daily reality. Despite international efforts, many families still lack access to basic needs, like food due to the disruption in supply chains, creating an urgent need for alternative food sources to meet survival needs. This situation is more acute in countries like South Sudan, where floods leave them with no arable land and no livestock (5) and people have resorted to eating water lily bulbs during periods of food scarcity. In other settings, such as Sudan and Gaza, severe food insecurity has forced some populations to rely on highly inadequate food sources for survival, while infants continue to experience acute malnutrition and, in some cases, starvation despite ongoing international humanitarian assistance (6).

Emergency therapeutic food supplies are highly sensitive to political pressures, both for donors and recipients. When these supplies do not reach affected populations, millions are left vulnerable to starvation. These systemic failures and the growing susceptibility to disasters highlight the urgent need for innovative, scalable solutions tailored to the contemporary challenges of LMICs. In this scenario, the development of local, sufficient, affordable, nutritious, and safe food sources is essential to ensure nutritional resilience and prevent malnourishment, starvation, famine, and mortality (7).

In this respect, we propose duckweed, one of the fastest-growing aquatic plants, as a promising candidate for famine alleviation, due to its high protein and amino acid content, global adaptability, and easy cultivation (8). Often considered a pest, it thrives in slow, stagnant waters and requires no fertile or arable land, making it a resource-efficient food source that helps overcome logistical barriers in humanitarian crises. Back in 1992 its nutritional potential had been first elucidated, with a study demonstrating its suitability as a feed ingredient and its capacity to support normal or compensatory growth in broiler chicken in Peru, highlighting its potential as a feed source (9).

This review aims to evaluate duckweed’s suitability as a famine food in LMICs and humanitarian crisis settings by addressing three critical dimensions. First, its nutritional adequacy is assessed through an examination of its macro- and micronutrient composition, with special emphasis on protein and essential amino acid profile, to determine whether it meets the requirements to support human health and development. Second, its safety is examined through a review of antinutritional factors, pathogen contamination, and heavy metal accumulation, alongside strategies to mitigate these risks. Finally, cultural perceptions of duckweed consumption are discussed, recognizing their importance as a determinant of feasibility in real-world humanitarian contexts.

## 2 Methodology

The search strategy focused on the suitability of duckweed for human consumption. To explore the potential of duckweed as a food source, we conducted searches in peer-reviewed literature databases using relevant terms and synonyms in English, including *W. globosa*, *W. arrhiza*, *L. minor*, human consumption, digestibility, heavy metal accumulation, and sun-drying microbes. Databases consulted included PubMed, Scopus, Web of Science, and JSTOR. No restrictions were placed on the date of publication, and articles were limited to those published in English. Peer-reviewed articles, randomized controlled trials, and technical reports were manually screened to exclude studies not related to the topic. Studies were included if they addressed duckweed as a human food source, its nutritional value, food safety, drying methods, consumption techniques, or acceptability. Relevant articles were then examined in further detail to determine whether they aligned with the scope of this review. Studies were not considered if their primary research question fell outside the scope of duckweed as a human food source, for example, work focusing on duckweed for fuel or wastewater treatment without linking it to human food value. As this review relied exclusively on publicly available secondary data, ethical approval was not required.

## 3 General characteristics of duckweed

Duckweeds are small free-floating aquatic plants belonging to the family *Lemnaceae*, which comprises five genera: *Spirodela*, *Landoltia*, *Lemna*, *Wolffiella*, and *Wolffia*, with a total of 36–37 recognized species (10, 11). Among these species, *Wolffia globosa*, *Wolffia arrhiza*, and *Lemna minor* have been the most studied for human consumption due to their high protein content, favorable amino acid composition, and micronutrient density. Additionally, duckweed grows rapidly in bodies of water, doubling in biomass for approximately 4–5 days (12, 13). It stands as one of the fastest-growing plants on earth and one of the most efficient crops for biomass production (Table 1).

**TABLE 1:** Comparative growth rates of *Lemnaceae* species and rice.

Crop	Specific growth rate per day (%)	Daily productivity\(g/m^2^/day)	Doubling biomass (number of days)	Harvesting time (number of days)
Rice	6.42*	19.71	–	90–180
*W. globosa*	14.62–15.33	97.76–176.66	4–5	5
*L. minor*	17.57	35.00–72.92	4–5	2–3

Table data compiled from Said et al. (12), Sembada and Faizal (13), and Driever et al. (76).

*Relative Growth Rate for Oryza sativa L. (77) (No information was found for *W. arrhiza*).

This aquatic plant can be harvested every 2–3 days using 90% less water per kilogram of protein produced than land-based crops. For instance, rice, the world’s most widely consumed staple, requires approximately 90 to 180 days to reach maturity and yields around 10–15 tons of above-ground biomass per hectare per growing season under optimal conditions, which translates to 4.22 g/m^2^/day in the best-case scenario (14). Compared to duckweed’s productivity of 72.92 g/m^2^/day, the disparity highlights duckweed’s exceptional productivity and efficiency, while reducing freshwater withdrawals, and freeing hectares of land.

It thrives in warm, well-lit environments. The optimum growth temperatures range from 20 to 35 °C, but it tolerates temperatures as high as 40 °C or as low as 3 °C, although its growth rate may decrease (15). It can tolerate a wide pH range of 3.5–9.0 as well as low levels of light. Thus, it is very adaptable to diverse environments, and it is widely distributed across temperate and tropical regions, highlighting its potential as sustainable food sources in diverse famine-prone regions.

Nutrient-rich waters, particularly those containing high nitrogen and phosphorus concentrations (13, 16, 17) are ideal for duckweed’s proliferation, as reflected in enhanced relative growth rates, accelerated biomass accumulation, and effective nutrient assimilation. Importantly, the growth-medium composition will not only determine the plant’s concentration of macro- and micronutrients but it will also modulate the accumulation of antinutritional factors, pathogenic contaminants, and heavy metals present within the plant’s biomass, which will be further evaluated with respect to consumption safety in subsequent sections (18).

Harvesting *L. minor* is relatively straightforward due to its larger frond size. It can be efficiently skimmed using a fine mesh net and transferred to a perforated container or mesh bag to facilitate drainage. In contrast, *W. globosa* and *W. arrhiza* are significantly smaller, requiring the use of ultra-fine mesh nets or specialized filtration fabrics to ensure efficient harvesting.

## 4 Nutritional composition of duckweed

Duckweeds have notable levels of protein, fiber, and phytonutrients. Certain species resemble animal protein, with a richer array of essential amino acids than most vegetable proteins (19). As shown in Table 2, *W. globosa* can reach 48.2% protein of Dry Weight (DW) under optimal growth conditions, which compares favorably to Plumpy’Nut, a peanut-based packed ready-to-use therapeutic food used to treat severe acute malnutrition in humanitarian settings. Likewise, *W. arrhiza* and *L. minor*, can reach up to 50.89% and 39.07% of protein DW, respectively, and demonstrate a substantial amino acid profile (13). Levels of essential amino acids frequently reach concentrations that meet or even surpass the recommended intake of FAO/WHO (20, 21) as shown in Table 3.

**TABLE 2:** Comparative nutritional composition of *Lemnaceae* species, and Plumpy’Nut.

Components	*W. globosa*	*W. arrhiza*	*L. minor*	Plumpy’Nut
Protein (%)	26.76–48.20	19.80–50.89	7.50–39.07	6.63
Fat (%)	0.49–9.60	2.43–6.07	1.95–2.32	18.48
Crude fiber (%)	8.80–16.76	13.30–14.72	9.17–12.24	–
Ash (%)	7.68–20.43	11.71–18.30	11.75–23.71	1.20*
Carbohydrate (%)	36.73–48.31	31.33–43.60	54.00–58.07	23.91

Expressed as a range (%) of dry weight. Table data compiled from Said et al. (12), Hu et al. (21), Chakrabarti et al. (28), Hanczakowski et al. (30), Bhanthumnavin and Mcgarry (70), Ruekaewma et al. (71), Boonarsa et al. (74), Obinna Ben et al. (78), Yılmaz and Günal (79), Rusoff et al. (80), Maciejewska-Potapczykowa et al. (81), Jairakphan (82), Chantiratikul et al. (83), and Sirirustananun (84).

*Estimated from the sum of all minerals.

**TABLE 3:** Amino acid profiles of *Lemnaceae* species vs. WHO/FAO requirements for human nutrition.

Amino acid (mg/100 g of protein)	*W. globosa*	*W. arrhiza*	*L. minor*	FAO/WHO requirements
Histidine*	989	990	559	1,600
Isoleucine*	1,373	2,762	1,281	1,300
Leucine*	2,664	4,503	2,414	1,900
Lysine*	1,457	3,434	6,792	1,600
Methionine*	418	1,151	519	1,700
Phenylalanine*	1,523	2,679	1,597	1,900
Threonine*	782	2,587	1,209	900
Tryptophan*	639	556	365	500
Valine*	1,770	3,138	1,655	1,300
Alanine	2,303	4,525	1,815	–
Arginine	2,124	3,578	3,060	–
Aspartic acid	3,469	9,763	2,405	–
Cystine	1,859	104	381	–
Glutamic acid	2,874	8,273	3,822	–
Glycine	1,352	2,730	1,650	–
Proline	1,183	1,672	1,248	–
Serine	1,014	2,812	1,410	–
Tyrosine	1,211	3,381	1,096	–

Table data compiled from Sembada and Faizal (13), Hu et al. (21), Chakrabarti et al. (28), Ruekaewma et al. (71), Boonarsa et al. (74), Yılmaz E and Günal (79), Rusoff et al. (80), Jairakphan (82), Duangjarus et al. (85), and Siriwat et al. (86).

*Essential amino acids.

Duckweed also accumulates minerals efficiently as demonstrated in Table 4. Notably, iron levels (1.6–3.7 mg/g DW) surpass spinach (2.6 mg/g) (22). Magnesium, another abundant accumulated mineral in *W. globosa*, is comparable to those found in pumpkin seeds, quinoa, avocado, or salmon. Similarly, potassium and sodium concentrations are found to be high, contributing to the plant’s nutritional value.

**TABLE 4:** Micro and macronutrient profiles of duckweed genera.

Minerals	Units of measurement	*W. globosa* (FDW)	*W. arrhiza* (FDW)	*L. minor* (FDW)	RUTF guidelines by WHO*
Iron	(mg/kg)	100.00–290.00	160.00–290.00	–	100.00–140.00
Manganese	(mg/kg)	203.00–302.00	78.40–264.00	–	–
Copper	(mg/kg)	2.32–4.43	2.70–3.31	–	14.00–18.00
Zinc	(mg/kg)	38.00–84.20	22.50–52.68	–	110.00–140.00
Iodine	(mg/kg)	0.20–0.50	0.20–0.39	–	0.70–1.40
Selenium	(mg/kg)	< 0.03	< 0.03	–	0.20–0.40
Calcium	(g/kg)	13.40–25.20	19.80–25.70	–	3.00–6.00
Phosphorus	(g/kg)	13.50–17.10	16.50–18.80	–	3.00–6.00
Sodium	(g/kg)	0.11–0.30	0.12–0.38	–	2.00–4.00
Potassium	(g/kg)	42.10–81.57	50.43–93.70	–	11.00–14.00
Magnesium	(g/kg)	1.96–3.17	2.39–3.06	–	0.80–1.40

Table data compiled from Appenroth et al. (18) and WHO (87). FDW, freeze dry weight (No comparable information was found on *L. minor*).

*WHO guideline on the dairy protein content in ready-to-use therapeutic foods for treatment of uncomplicated severe acute malnutrition (Plumpy’Nut values vary slightly by manufacturer batch).

Beyond minerals, *W. globosa* is also rich in other bioactive compounds. Monthakantirat et al. (23) found that the ethanolic extract of *W. globosa* contains significant levels of flavonoids, phenolics, and carotenoids. The total flavonoid content was 38.99 ± 0.44 mg/g of the extract; the phenolic content reached 40.83 ± 4.99 mg/g extract; and 2.52 ± 0.10 mg/g of β-carotene. It has also been reported that the B12 level was stable, regardless of the seasonal or environmental conditions, such as water temperature or light hours, and that it is reliable in replacing red meat (24). Similarly, the content of flavonoids in *W. arrhiza* (41.2 ± 0.13 mg/g FW) was much higher than those in most common edible vegetables and fruits, such as bitter melon (0.15 mg/g FW) and berries (0.19–1.27 mg/g FW) (21, 25, 26).

Furthermore β-sitosterol and stigmasterol has been identified in *W. globosa*, which provides anti-inflammatory properties and cardiovascular benefits (27). Additionally, the sterols in *W. globosa* were found to inhibit nitric oxide production in macrophage cells. This suggests that the sterols in *W. globosa* have protective effects against chronic inflammation. For *W. arrhiza*, a significant portion of its fatty acids were polyunsaturated, comprising 44.46% polyunsaturated fatty acids. Among these, omega-3 fatty acids were prominent, accounting for 22.17% (13). *L. minor* fatty acid profile is also favorable, with polyunsaturated fatty acids comprising 60%–63% of total fatty acids, including significant levels of α-linolenic acid and linoleic acid (28).

Figure 1 presents a comparative radar chart of the mean nutritional composition derived from Table 2, illustrating the distinct nutritional profiles of the three *Lemnaceae* species alongside Plumpy’Nut. Among the three species, *W. globosa* may be the most balanced candidate. It has a very high protein content and satisfies the FAO/WHO sulfur amino acid requirement when methionine and cystine are scored together. It also has the most thoroughly documented micronutrient and bioactive profile. *W. arrhiza* contains very high protein quantity and exhibits the highest absolute concentrations across most essential amino acids. However, its critical limitation is a near-absence of cystine, which leaves it deficient in total sulfur amino acids despite its abundant methionine, lowering its overall protein quality by FAO scoring. *L. minor* contains the lowest protein content and the lowest sulfur amino acid total. It is the most extensively studied species for phytoremediation, and is widely used as animal feed, but its lower protein quality and sparse micronutrient documentation make it a weaker candidate for direct therapeutic application.

**FIGURE 1:**
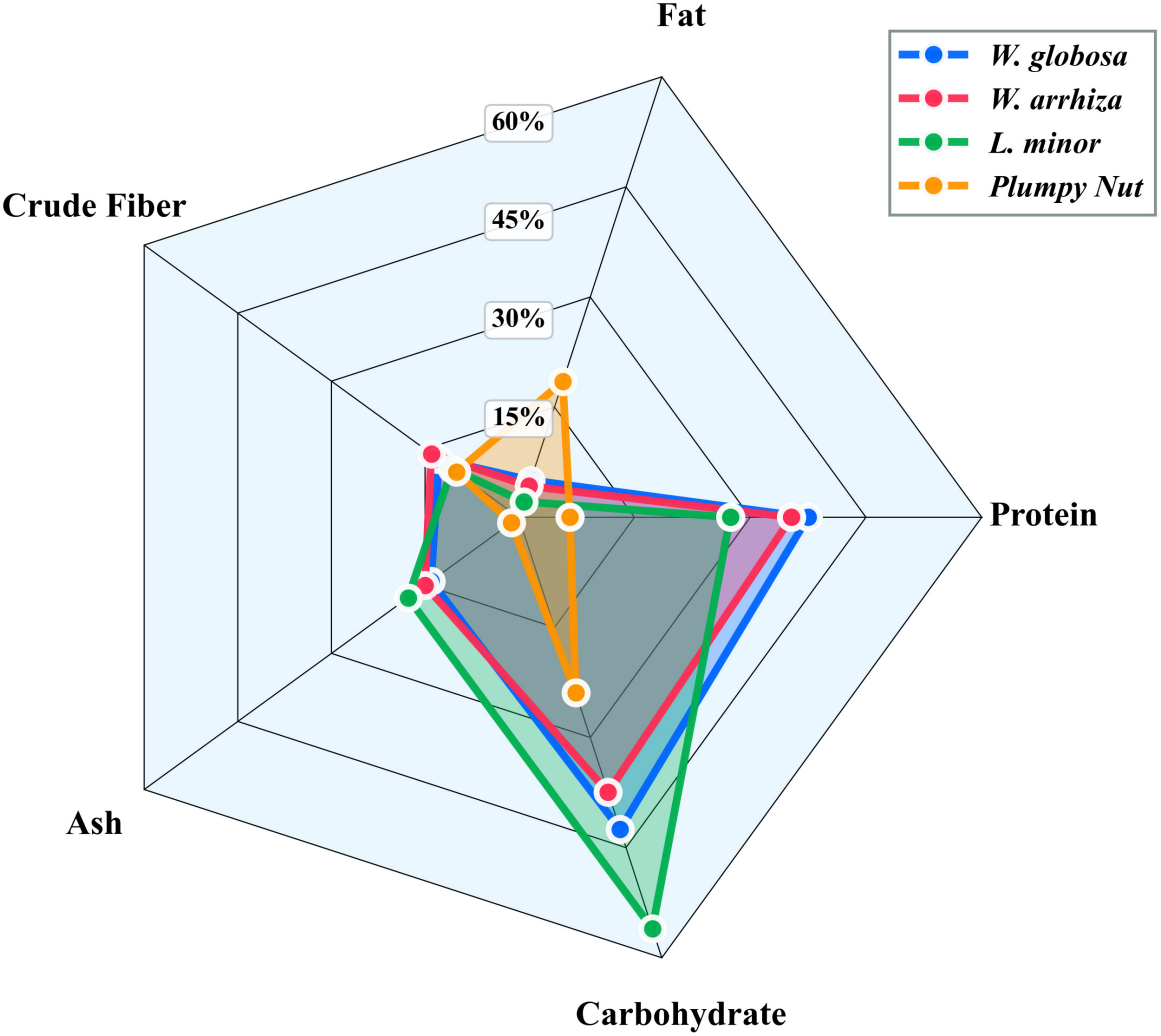
Nutritional composition of *Lemnaceae* species versus Plumpy’Nut.

Overall, duckweed, especially the *Wolffia* species, are outstanding high-protein, iron-rich, locally producible ingredients. However, based on WHO RUTF criteria, they may not be a standalone therapeutic food, as all three species tend to be energy-poor, with fat contents below that of Plumpy’Nut, which is critical for acute malnutrition, to regain a large amount of energy quickly and in minimum mass volume.

## 5 Antinutritional factors (ANFs), pathogen contamination, heavy metals, and remediation techniques

The aquatic-based nature of duckweed makes it sensitive to both endogenous (produced by them naturally or during processing) and exogenous (absorbed by the culture media and the environment) risks (29). Thus, the nutritional value, antinutritional factors (ANFs), pathogen load, and heavy metal concentrations in duckweed vary significantly depending on both the species and the growth medium. For instance, while swamps and sewage offer nutrient-rich conditions that support rapid growth and high nutrient density, they also present heightened risks of contamination, including pathogens, heavy metals, and other toxic substances (30), which will be discussed in this section.

### 5.1 Antinutritional factors (ANFs) in duckweed

Several antinutritional compounds have been identified in duckweed species and quantified across studies, including oxalates, phytates, tannins, and other secondary metabolites of potential nutritional relevance.

Oxalic acid and oxalates range from 181–219 mg/100 g dry weight in *L. minor*, 230–637 mg/100 g in *W. globosa*, and up to 804 mg/100 g in *W. arrhiza* (8, 21, 31–33). Although oxalates can reduce calcium bioavailability and contribute to kidney stone risk, *Wolffia* species appear to lack calcium oxalate crystal formation, which is the form most strongly associated with adverse health effects.

Phytic acid concentrations vary from below detection (< 140 mg/100 g dry weight) in *W. globosa* and ∼22 mg/100 g in *W. arrhiza* to 732.84 mg/100 g in raw *L. minor*, with substantial reductions following processing (∼166 mg/100 g) (8, 21, 32, 33). Overall, these levels are considered unlikely to meaningfully impair mineral absorption under typical dietary conditions.

Tannin concentrations range from 64 mg/100 g dry weight in *L. minor* to 983 mg/100 g in *W. arrhiza* and 1,720–2,000 mg/100 g in *W. globosa* (20, 21, 34, 35). Despite higher values in *Wolffia*, evidence from human studies indicates no significant impairment of essential amino acid bioavailability.

Saponins are absent or unreported in *Wolffia* species and are present in *L. minor* at approximately 0.72% dry weight, with substantial reduction after cooking (36). Hydrogen cyanide is not detected or not reported in *Wolffia*, while *L. minor* contains low levels (∼4.9 mg/100 g dry weight) that decrease with processing (36).

Trypsin inhibitors are not quantitatively characterized in any species, but available evidence suggests low levels in *Lemna* and even lower levels in *Wolffia* compared with legumes (34, 37). Lectins or hemagglutinins are not meaningfully detected in any of the species, and protein analyses show no relevant similarity to known dietary lectins or allergenic proteins (34, 38).

Although, a European regulatory assessment has raised concerns regarding increased manganese exposure from duckweed-derived protein-powders, this issue is primarily associated with concentrated preparations such as protein-isolates used at high inclusion levels in processed foods (39). However, in whole-plant forms of *Lemna* or *Wolffia*, manganese concentrations are considerably lower and closer to those observed in conventional leafy vegetables.

Overall, antinutritional factors in duckweed remain within ranges generally considered acceptable for human consumption, particularly when standard processing methods such as cooking are applied.

### 5.2 Pathogen contamination

In resource-limited settings, particularly during famine or humanitarian crises, the cultivation of duckweed would most likely occur in untreated or contaminated water sources. Such environments frequently contain pathogenic microorganisms or parasites, which can be readily absorbed by duckweed during growth. Duckweed grown in wastewater or fecal-contaminated waters can harbor *Escherichia coli* and *Salmonella* (40). Chlorine-resistant parasites such as *Cryptosporidium* may also be present and thus pose serious risks to malnourished infants (e.g., dehydration and worsening nutrient deficits) (41, 42).

Studies have shown that the microbial contamination levels in these species are largely dependent on their growth environment (43). When cultivated under hygienic conditions, duckweed species pose no significant concerns regarding genotoxicity. While naturally occurring bacteria and fungi can be present, the risk of harmful pathogens such as *E. coli*, *Salmonella* spp., and *Clostridium perfringens* remain low when cultivation practices adhere to sanitary protocols. A controlled aquatic environment significantly reduces the uptake of hazardous contaminants, which ensures that the final product remains a safe and nutritious food source (44).

In addition to preventive measures such as hygienic cultivation, context-appropriate inactivation methods can further reduce microbial load in humanitarian crisis settings. Duckweed exposure to sunlight and desiccation inactivates many pathogens, including bacteria, parasites, yeast and mold, extending its shelf life. For instance, Sinton et al. (45) reported that direct sunlight accelerated the inactivation of *Campylobacter jejuni*, *Salmonella enterica*, and *E. coli* in aquatic environments, with pathogen die-off rates significantly higher under full-spectrum solar radiation than in shaded or dark conditions.

Likewise, Bhila et al. (46) demonstrated that sun-drying significantly reduced the levels of coliforms, total bacterial count, yeast, and molds both in cabbage and beetroot leaves. However, there was no significant difference in *E. coli* levels between wet and dried samples, which suggests that while sun drying is effective in lowering the overall microbial load, it may not guarantee the elimination of some pathogens. Still, he recognized sun-drying as an acceptable method for use by small scale farmers, especially for preservation purposes. Survival studies on *Salmonella* and Shiga toxin producing *E. coli* (STEC) show that their viability declines markedly over time when crops are stored at ambient temperatures, with complete inactivation reached after 35 to 70 days of desiccation under low-moisture conditions (47).

These studies collectively demonstrate the efficacy of solar-based and dry-storage techniques in reducing microbial contamination in plant-based foods. These techniques present a low-cost and effective method particularly well-suited for resource-limited settings (48). The approach is especially advantageous in tropical regions, where high solar intensity and prolonged dry conditions provide optimal environmental parameters both for effective solar drying and abundant duckweed biomass production. Traditionally employed for food preservation, solar drying extends shelf life by preventing spoilage, while also reducing moisture content. This dehydration facilitates easier handling and lowers transportation costs due to the reduced weight of the preserved material.

However, Singh and Sorokhaibam (49) noted some disadvantages of direct solar drying. When exposed to sunlight, the harvested food is often associated with risks such as insect infestation, contamination from dust and dirt, and prolonged, uncontrolled drying periods. A particularly critical concern arises during nighttime; if sun-dried fruits are left uncovered or exposed, the cool, humid night air can condense around the plant, reintroducing moisture and thereby prolonging the drying process. Additionally, research has shown that, for *W. globosa*, and thus likely to all *Lemnaceae* species, the iron, potassium, magnesium, zinc, and sodium content is considerably higher in its wet form compared to dry matter. This difference is likely attributed to the heating effect during the drying process, which causes minerals to escape or vaporize (23).

Yadav et al. (50) examined mechanical rupture and boiling as methods to enhance nutritional and functional qualities of *W. globosa*. Mechanical rupture was one of the most effective methods to preserve the highest phenolic and flavonoid content, enhancing antioxidant properties and protein digestibility (50). While boiling did not outperform, it is still a good alternative, as it improves protein accessibility and reduces microbial contamination.

### 5.3 Heavy metal contamination

The European Food Safety Authority (EFSA) evaluated fresh *Wolffia globosa* plants and authorized their sale as a traditional food for human consumption, noting that when cultivated in hygienic, controlled water, heavy metals and cyanotoxins remain below health-risk thresholds (39). EFSA also reported that cyanobacterial toxin concentrations and estimated daily intakes were well within tolerable limits, even for vulnerable populations such as infants. Nevertheless, EFSA emphasized that *W. globosa* is highly sensitive to the chemical composition of its growth medium. This finding highlights the need for careful monitoring of cultivation water to ensure safety.

Several duckweed species have been extensively studied for phytoremediation due to their ability to accumulate heavy metals (29). While the most commonly used species for phytoremediation belong to the *Landoltia* and *Lemna* genera, the accumulation by *Wolffia* species should be evaluated to assess its safety as food source. Reported sorption uptake rates indicate substantial species-dependent variability, including cadmium (0.2–7.7 mg/g dry weight), chromium (0.6–1.2 mg/g), cobalt (up to 21 mg/g), copper (1–5.5 mg/g), lead (0.28–10 mg/g), zinc (0.8–20 mg/g), arsenic (0.5–2.2 mg/g), and nickel (5.5–12.9 mg/g) (29, 51–54).

Depending on the growth medium contamination, heavy metal levels could exceed WHO safety thresholds for infant foods. This is a potential concern to acute and chronic health, such as neurological development, kidney dysfunction, and osteoporosis (55–57).

Nevertheless, there are some potential water remediation techniques to apply before introducing duckweed into the water bodies to alleviate the heavy metal concentration. Research has shown that applying biochar, a type of charcoal made from low-oxygen-heated organic material, to stagnant water systems effectively reduces heavy metal concentrations. Biochar has high surface area, porosity, and an abundance of functional groups that make it capable of binding metal ions. When introduced into contaminated aquatic environments, biochar adsorbs metals such as lead (Pb), cadmium (Cd), and arsenic (As), decreasing their bioavailability and toxicity. Thus, biochar has been found to be an effective and low-cost remediation strategy suitable for resource-limited settings (58–62). Plants cultivated in biochar-treated water exhibit improved biomass and reduced metal accumulation, which supports ecological restoration and safe biomass production.

Similarly, the application of cow dung and sawdust to stagnant waters has demonstrated significant potential for heavy metal remediation. Both materials function as natural biosorbents due to their rich organic content and surface functional groups capable of binding metal ions. Cow dung contains humic substances and microbial populations that facilitate the adsorption and transformation of toxic metals such as lead (Pb), cadmium (Cd), and chromium (Cr). On the other hand, sawdust, particularly from hardwoods, offers a high lignocellulosic content with hydroxyl and carboxyl groups that effectively sequester metal contaminants (37, 63, 64).

As duckweed itself functions as a phytoremediator, an initial batch can be cultivated and harvested solely for water purification purposes. Once the water quality improves, a second batch could theoretically be grown in the treated environment, thereby reducing the risk of heavy metal accumulation in duckweed intended for feed or food use. However, this approach remains speculative and would require empirical validation, as residual contaminants and recontamination from decaying plant material may still pose risks.

## 6 Digestibility and bioavailability

A randomized controlled trial by Kaplan et al. (35) reported a Protein -Digestibility-Corrected Amino-Acid-Score (PDCAAS) of 89% in a cultivated strain of *W. globosa*. Participants consuming iso-protein (30 g) *W. globosa* meals showed significant increases in blood concentrations of seven EAAs (except lysine and methionine) within 3 h, similar to cheese and peas, which increased six EAAs. Additionally, *W. globosa* elevated serum vitamin B12 levels significantly compared to both cheese and peas, a rare trait for plant-based proteins (35).

Hu et al. (21) calculated *in vitro* digestibility of essential amino acids (EAAs) in *W. arrhiza*. They were found to be high, with most EAAs exceeding 90% digestibility (21). This high digestibility significantly contributes to the protein quality of *W. arrhiza*, as reflected in its Digestible Indispensable Amino Acid Score (DIAAS) of 0.75, categorizing it as a good-quality protein higher than wheat which had 0.40 and peas, a DIAAS of 0.64 (21).

Furthermore, Yaskolka Meir et al. (65) demonstrated in a 6-month trial that a Green-Mediterranean diet supplemented with *W. globosa* maintained iron homeostasis in 294 non-anemic individuals with abdominal obesity and dyslipidemia. Compared to control groups, the *W. globosa*-supplemented group showed significant increases in hemoglobin (0.23 g/dL vs. −0.1 g/dL, *p* < 0.001), serum iron (8.21 μg/dL vs. −5.23 μg/dL, *p* < 0.05), and transferrin saturation (2.39% vs. −1.15%, *p* < 0.05), along with a rise in folic acid levels (*p* = 0.011). They performed a complementary animal study on iron-deficient anemic rats demonstrating that *W. globosa*-derived iron was as bioavailable as ferrous gluconate, effectively restoring hemoglobin levels in anemic models (65).

Also, Zelicha et al. (66) conducted a randomized controlled crossover trial to compare the postprandial glycemic response to meals containing *W. globosa*, versus yogurt shakes. The postprandial glucose peak was lower for the *W. globosa* shake compared to the yogurt shake, the peak occurred later and returned to baseline faster, indicating a slower and more controlled glucose response. Fasting glucose levels were also lower in the *W. globosa* category the next morning. This suggests that *W. globosa*, due to its high fiber and polyphenol content, may offer a beneficial alternative to dairy-based shakes, potentially supporting better glycemic control.

Seephua et al. (67) investigated the *in vitro* effect of incorporating *W. globosa* powder at concentrations of 1, 3, and 5% into rice noodles, finding that the 5% formulation doubled protein content and improved protein digestibility by 22% compared to the control. Starch digestibility was also favorably affected, with increases in resistant and slowly digestible starch fractions and a reduction in estimated glycemic index, suggesting a lower glycemic response (67).

Regarding *L. minor*, a randomized cross-over trial with twelve healthy adults compared the protein digestibility of *L. minor* and green peas (68). Peak postprandial plasma amino acid concentrations were lower following duckweed consumption than after peas (3,014 vs. 3,429 μg/ml for total amino acids), indicating reduced protein digestibility in whole-plant form. Glucose and insulin responses were also lower (68). However, no adverse physiological and gastrointestinal effects were reported ensuring its human consumption safety (68).

In 2025, Ziêæ et al. (69) conducted a critical review of the nutritional and functional potential of various duckweed species as a food ingredient, concluding that these aquatic plants demonstrate a favorable amino acid profile, high protein digestibility, and low allergenic potential. That study highlighted its antioxidant, anti-inflammatory, glycemic-modulatory, prebiotic, and iron-bioavailability properties, suggesting therapeutic relevance as next-generation plant protein (69).

## 7 Cultural perceptions and dietary practices

The integration of *Wolffia* species into traditional diets, particularly in Southeast Asia, is well documented. *W. arrhiza* and *W. globosa* have long been consumed in countries such as Myanmar, Laos, and Thailand, where they are commonly sold in local markets as edible greens (70–73). These species are colloquially referred to as Khai-nam, Mankai, Khai Phum, Khai Nhae, “water-eggs,” “watermeal,” or “poor people’s food,” and are used in various local dishes (71).

Their sustained culinary presence reflects nutritional value, cultural acceptance, and local recognition (23). Among these, *W. globosa* is particularly notable for its high protein and fiber content, which has been widely acknowledged within Thai food culture. It is frequently available in markets and has been promoted for large-scale cultivation in Thailand (70–74). *L. minor* is commonly known as common duckweed, adas almay, or qing ping. While it has not been deeply incorporated into culinary traditions, it has been recognized for its applications in traditional medicine and pharmaceutical contexts, as antipruritic, antiscorbutic, astringent, depurative, diuretic, febrifuge and soporific (75).

Overall, duckweed’s use across diverse regions demonstrates its long-standing cultural adaptability and nutritional relevance. However, its uptake remains highly context-dependent, shaped by local ecological conditions, cultural perceptions, and the availability of alternative food sources. These factors will ultimately determine the feasibility of introducing duckweed as an acceptable and sustainable famine food in new settings.

## 8 Conclusion

This review synthesizes evidence supporting the nutritional potential of the duckweed species *W. globosa*, *W. arrhiza*, and *L. minor*, as novel therapeutic foods in famine-affected LMICs and humanitarian crises. Across duckweed species, protein content reaches up to 48.2% DW in *Wolffia globosa*, 50.9% DW in *Wolffia arrhiza*, and 39.1% DW in *Lemna minor*, while also providing a comprehensive essential amino acid profile. Protein quality is considered high, with PDCAAS values reaching 89% for *W. globosa* and a DIAAS of 0.75. Additionally, a diverse micronutrient profile, including bioavailable iron, zinc, vitamin B12, and various immunomodulatory phytochemicals, reinforces the nutritional utility in contexts lacking dietary diversity. High biomass productivity (up to 72.92 g/m^2^/day), water efficiency, and broad climatic tolerance indicate production scalability.

However, two critical limitations preclude direct substitution for standard RUTFs, such as Plumpy’Nut. First, the low lipid content (0.5%–9.6% DW) results in an energy density insufficient for the rapid caloric repletion required in severe acute malnutrition treatment. Second, aquatic cultivation introduces risks of exogenous contamination. Specifically, the capacity of these species to bioaccumulate heavy metals and other contaminants from culture media presents a pathway to toxicity, particularly for malnourished pediatric populations whose compromised gut integrity and developing neurological systems amplify susceptibility. Its realistic role may require fortification and complementation with a lipid source premix.

Provided that cultivation water is monitored to prevent heavy metal accumulation, evidence supports the deployment of duckweed as a high-protein, micronutrient-dense dietary supplement. At the least, given its remarkable rapid growth capacity achieved with minimal input and labor, it should be considered an intermediate intervention capable of attenuating malnutrition and micronutrient deficiencies while conventional supply chains are re-established. This framing is clinically and logistically realistic. In famine contexts, where therapeutic food access is interrupted by conflict, flooding, or blockade, a locally cultivable, rapidly renewable protein source requiring minimal infrastructure offers a meaningful bridge intervention to prevent people from starving. Antinutritional factors remain comparable to common vegetables, and low-cost, field-deployable decontamination strategies are available to mitigate microbial loads.

These findings must be interpreted with caution as the current literature exposes substantial inter-study variability, potentially arising from (1) unspecified and divergent cultivation conditions, (2) lack of quality control during cultivation, (3) inconsistent biomass quantification metrics, and (4) differences in nutrient availability across wild and semi-controlled or controlled systems, collectively undermining direct comparative analysis. Results regarding duckweed cultivation failures or adverse nutritional outcomes are largely absent from the current literature. Additionally, most of the evidence arises from laboratory analysis, in controlled environments and animal models, as opposed to analysis in child and adults, and especially in malnourished patients, who are the intended population.

Addressing current evidence gaps will require progress on three fronts. First, agronomic research is needed to refine cultivation techniques and to assess feasibility and suitability in LMICs, including the sensitivity of duckweed’s nutritional composition to growth-medium conditions. Second, randomized controlled trials in malnourished cohorts are needed to pragmatically assess its suitability for famine alleviation, to characterize nutrient bioavailability, and to establish operational conditions for deployment in humanitarian emergencies. Third, its adoption as a food source would require regulatory approval and a degree of standardization in production.

Context-specific risk-benefit assessments evaluating water source characterization, heavy metal and contaminant profiling, as well as pathogen exposure must precede field-scale introduction. Ultimately, while not a replacement for structured humanitarian food systems, duckweed represents a scientifically valid and operationally feasible complement for malnutrition management in emergency contexts, as it yields exceptional protein, amino acids and micronutrients requiring minimal labor, minimal input and doubling its biomass in days.
